# Supplementary material for: Amplicon-guided isolation and cultivation of previously uncultured microbial species from activated sludge
Source: Appl Environ Microbiol. 2023 Dec 5;89(12):e01151-23. doi: 10.1128/aem.01151-23 (PMC10734543; doi:10.1128/aem.01151-23)
Supplement: Supplemental file 1 — Fig. S1 to S4. [file aem.01151-23-s0001.pdf]

**Supplementary Information for:**

# Amplicon-guided isolation and cultivation of previously uncultured microbial species from activated sludge

**Authors:** Maarten D. Verhoeven[a*] Per H. Nielsen[a] Morten K. D. Dueholm[a#]

**Affiliations:**

[a]Center for Microbial Communities, Department of Chemistry and Bioscience, Aalborg University, Aalborg, Denmark.

[#]Correspondence to: Morten Kam Dahl Dueholm, Center for Microbial Communities, Department of Chemistry and Bioscience, Aalborg University, Fredrik Bajers Vej 7H, 9220 Aalborg, Denmark; Phone: +45 9940 8508; Fax: Not available; Email: md@bio.aau.dk

*Present address: Maarten D. Verhoeven, Wageningen University & Research, Wageningen, Netherlands.

**Table of content:**

Page 2-5: Supplementary Figure S1-S4

## Supplementary Figures:

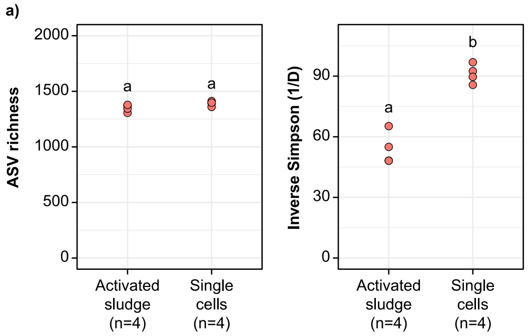


b)

| | Activated sludge | | | | Single cell preparation | | | |
|---|---|---|---|---|---|---|---|---|
| *Ca.* Phosphoribacter | 16.19 | 16.68 | 14.49 | 12.96 | 7.81 | 7 | 7.27 | 8.2 |
| *Rhodoferax* | 5.4 | 3.66 | 4.03 | 3.66 | 9.58 | 9.44 | 10.42 | 9.6 |
| *Propionicimonas* | 4.6 | 5.55 | 4.95 | 4.55 | 3.29 | 3.31 | 3.02 | 3.26 |
| *Trichococcus* | 3.95 | 4.53 | 5.21 | 4.96 | 3.17 | 3.39 | 3.35 | 2.83 |
| *Ca.* Microthrix | 5.32 | 5.67 | 5.72 | 5.22 | 1.49 | 1.48 | 1.45 | 1.4 |
| *Ca.* Brachybacter | 1.48 | 1.13 | 1.35 | 1.42 | 3.89 | 3.79 | 3.89 | 3.89 |
| *Rhodobacter* | 2.47 | 2.81 | 2.67 | 2.81 | 2.3 | 2.61 | 2.31 | 2.57 |
| midas_g_171 | 1.17 | 1.07 | 1.2 | 1.31 | 3.53 | 3.85 | 3.58 | 3.86 |
| *Acidovorax* | 1.61 | 1.27 | 1.21 | 1.4 | 3.13 | 3.25 | 3.35 | 3.48 |
| *Nitrospira* | 3.31 | 3.26 | 3.47 | 3.53 | 1.31 | 1.24 | 1.05 | 0.89 |
| midas_g_99 | 1.58 | 2.1 | 1.87 | 1.87 | 1.72 | 1.89 | 1.51 | 1.59 |
| *Hyphomicrobium* | 0.82 | 1.24 | 1.29 | 1.39 | 1 | 0.85 | 1 | 1.01 |
| *Terrimonas* | 1.02 | 0.6 | 0.74 | 0.63 | 1.34 | 1.18 | 1.23 | 1.15 |
| *Ca.* Epiflobacter | 0.77 | 0.4 | 0.67 | 0.52 | 1.45 | 1.35 | 1.41 | 1.25 |
| *Mesorhizobium* | 0.83 | 0.99 | 0.95 | 0.87 | 0.84 | 0.78 | 0.84 | 0.91 |
| *Sphingopyxis* | 0.62 | 0.6 | 0.61 | 0.64 | 1.02 | 0.96 | 1.01 | 1.07 |
| midas_g_882 | 0.79 | 0.78 | 0.77 | 0.68 | 0.83 | 0.71 | 0.78 | 0.81 |
| midas_g_731 | 0.78 | 0.72 | 0.67 | 0.69 | 0.82 | 0.85 | 0.65 | 0.86 |
| *Streptococcus* | 0.4 | 0.58 | 0.62 | 0.75 | 0.86 | 0.89 | 0.89 | 0.78 |
| *Ca.* Opimibacter | 1.02 | 0.63 | 0.87 | 0.69 | 0.61 | 0.65 | 0.67 | 0.6 |
| midas_g_70 | 0.96 | 0.66 | 0.7 | 0.58 | 0.71 | 0.71 | 0.63 | 0.73 |
| *Novosphingobium* | 0.5 | 0.54 | 0.59 | 0.52 | 0.8 | 0.95 | 0.81 | 0.94 |
| *Tessaracoccus* | 0.73 | 0.92 | 0.88 | 0.8 | 0.47 | 0.5 | 0.46 | 0.42 |
| *Devosia* | 0.55 | 0.5 | 0.45 | 0.5 | 0.78 | 0.79 | 0.75 | 0.83 |
| midas_g_49 | 0.6 | 0.9 | 1.01 | 0.85 | 0.4 | 0.38 | 0.4 | 0.45 |

**Figure S1**. Preparation of single cell suspensions from activated sludge. a) Alpha diversity based on 16S rRNA gene V1-V3 amplicon data. A two-sided t-test (Bonferroni correction, α=0.05) was used for pairwise comparison of individual groups and the results are shown with compact letter display (groups that do not share letters are significantly different. b) Heatmap of the 25 most abundant genera in the activated sludge and single cell suspensions. Genera marked in red lacks pure culture representatives. Figures are based on activated sludge collected the 3rd of March 2021 and used for anoxic cultivations.

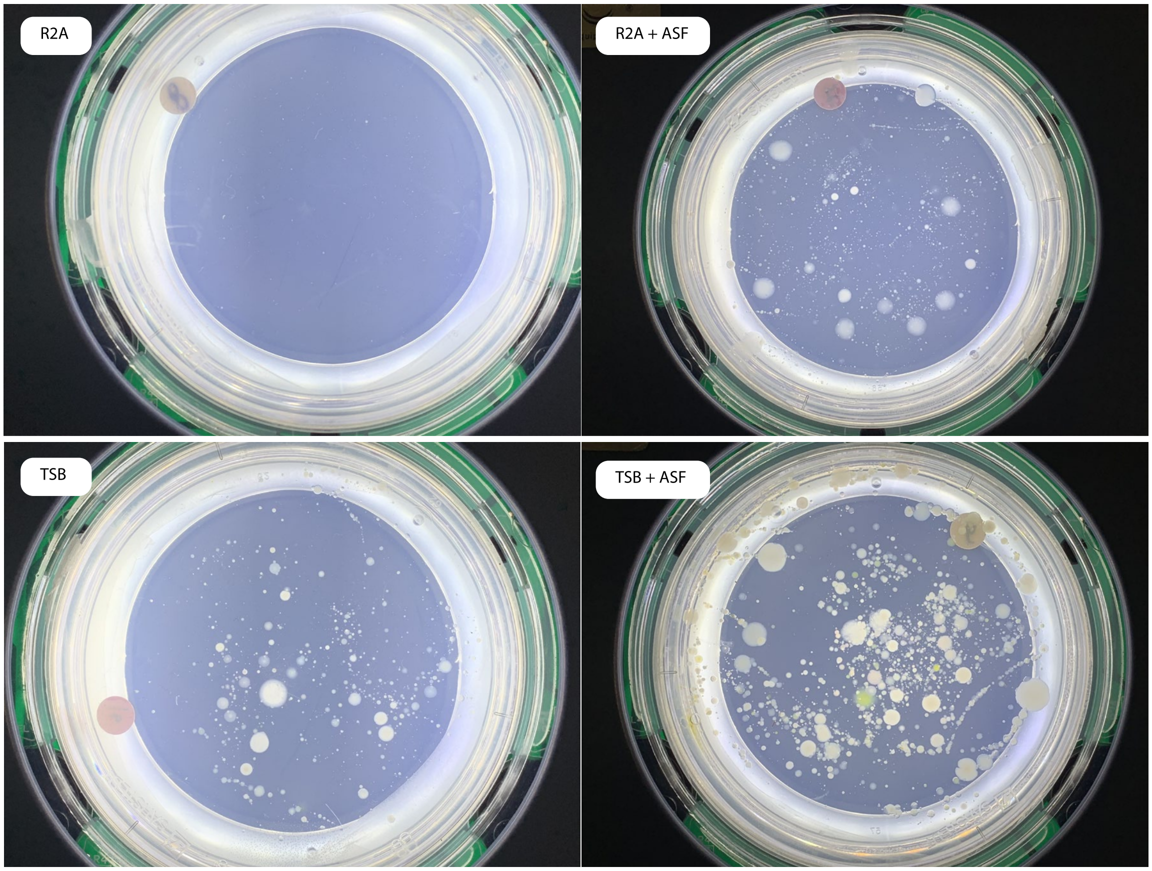

**Figure S2**: Photographs taken from agarose plates with for R2A and TSB medium with and without ASF. For each plate approximately 1000 cells were plated and incubated for two weeks at 25°C under oxic conditions.

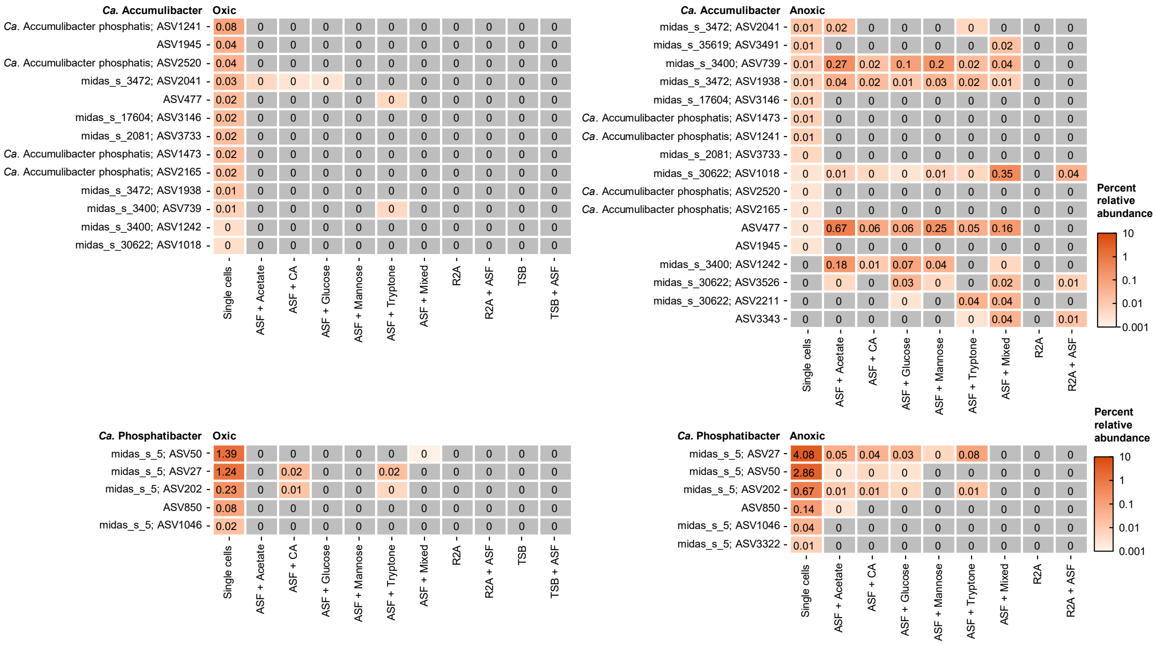


**Figure S3**. Heat map showing all *Ca*. Accumulibacter and *Ca.* Phosphoribacter ASVs found in the AS single cell suspension and the corresponding abundancy values found on each plate incubation under oxic and anoxic conditions. Values are the average abundance from four separately processed agarose plates. ASF: Activated sludge fluid; CA: Casamino acids.

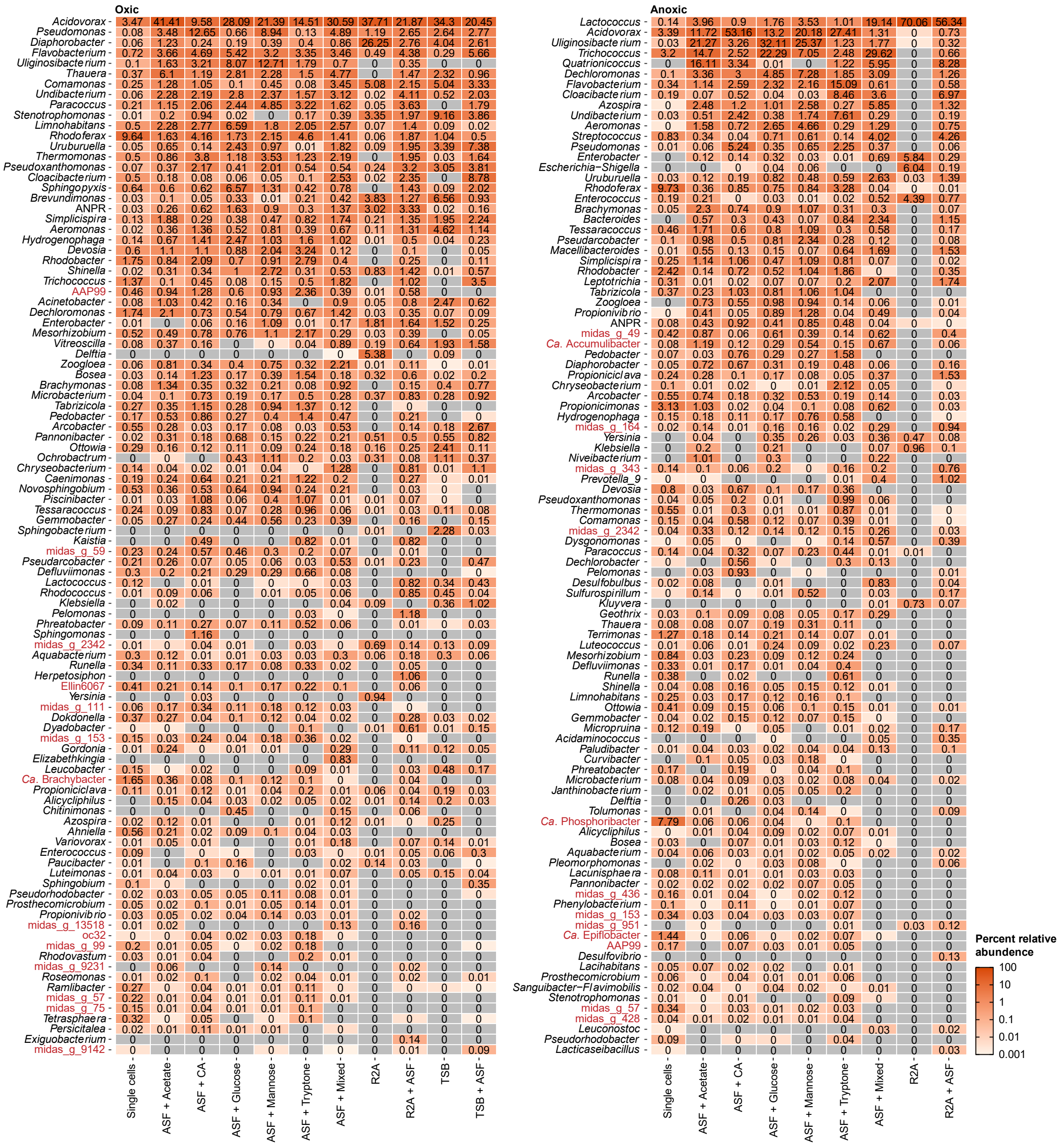


**Figure S4**. Heat map showing the top 100 genera found on the agarose plates after incubation under oxic and anoxic conditions. Values are the average abundance from four separately processed agarose plates, and genera are sorted based on average relative abundance across all media. Genera colored in red have no pure culture representatives. ASF: Activated sludge fluid; CA: Casamino acids; ANPR: *Allorhizobium-Neorhizobium-Pararhizobium-Rhizobium*.
